# A combination of bortezomib and rituximab yields a dramatic response in a woman with highly refractory immune thrombocytopenic purpura: a case report

**DOI:** 10.1186/1752-1947-8-19

**Published:** 2014-01-15

**Authors:** Namita Vinayek, Vivek Sharma

**Affiliations:** 1 University of Louisville, 529 South Jackson Street, Louisville, Kentucky 40202, USA

**Keywords:** Bortezomib, Chronic immune thrombocytopenic purpura, Rituximab

## Abstract

**Introduction:**

Chronic refractory immune thrombocytopenic purpura can be a challenging condition to treat. By definition, the standard first and second line treatments have failed in these patients and modalities such as thrombopoiesis-stimulating agents and more intensive immunosuppressive drugs are therefore used. However, there still remains a subset of patients who continue to be refractory to treatment.

**Case presentation:**

We present the case of a 30-year-old Hispanic woman with recurrent intracranial bleeds, in whom multiple lines of treatment had failed. She was treated with a combination of bortezomib and rituximab based on previously published data that suggested this therapy effectively blocks all antibody-producing cells. Our patient’s platelet counts rapidly improved and subsequently normalized following this treatment.

**Conclusion:**

To the best of our knowledge, this case represents the first report of the effective use of bortezomib and rituximab in highly refractory immune thrombocytopenic purpura. We believe further study of this therapy is warranted in this setting.

## Introduction

Immune thrombocytopenic purpura (ITP) is characterized by immune-mediated platelet destruction and is often a chronic disease in adults. Although most patients achieve a ‘safe’ platelet count with immunosuppressive therapy or splenectomy, about 5% develop severe, refractory disease, defined as a failure to respond to both splenectomy and rituximab. When such patients become symptomatic with clinically overt bleeding, their management can be quite challenging. Bortezomib is a proteasome inhibitor that functions by inhibiting the ubiquitin-proteasome system, key to cell functioning. Its role in hematological malignancies like multiple myeloma has been well documented. This proteasome-inhibiting property is now being investigated in human autoimmunity [1]. We report the case of a patient with refractory and symptomatic ITP in whom multiple lines of therapy had failed but who responded to a combination of bortezomib and rituximab, with normalization of her platelet count.

## Case presentation

A 30-year-old Hispanic woman with a history of ITP and autoimmune hemolytic anemia for almost 12 years presented with recurrent intracranial bleeds. She had developed chronic refractory disease following the failure of a splenectomy and rituximab therapy shortly after her diagnosis. Her hemoglobin level stayed between 8 and 10g/dL but she had severe thrombocytopenia with platelet counts typically in the range of 5000 to 10,000 cells/mm^3^. For the first seven years after diagnosis, she had experienced only intermittent minor bleeds, including epistaxis and menorrhagia. However, she then developed her first spontaneous intracranial hemorrhage, which was managed conservatively with platelet transfusions. Following that serious event, over the next four years she was treated with a variety of modalities in an attempt to raise her platelet counts to a safer level, including vincristine, cyclosporine, danazol, eltrombopag, romiplostim and rituximab as indicated in Table 1. She had little to no response to any of these agents, including rituximab, which was used on four different occasions (Table 1).

**Table 1:** Platelet trends, associated events and treatments given to our patient over 12 years

**Year**	**Platelet count (thousand/mm**^**3**^**)**	**Treatment**	**Events**
2001	10	Rho(D) + prednisone	Menorrhagia
2002	19 to 160	Splenectomy	Menorrhagia
2003	30 to 150	Rituximab (weekly ×4) + high dose prednisone	
2004	15 to 40	Danazol; rituximab (×2)	
2005	13 to 22	Vincristine (weekly ×4)	
2006	20 to 30	Observation	Hysterectomy
2007	10 to 12	High pulse decadron; rituximab×1; low dose cyclosporine 2.5mg/kg	Intracranial hemorrhage
2008	4 to 6	Intravenous immunoglobulin G; decadron; rituximab (weekly×4)	Ruptured ovarian cyst
2009	5 to 14	Eltrombopag 50mg to 75mg daily (×3 months)	
2010	9 to 12	Observation	
2011	6 to 15	Observation	
2012	4 to 10	Intravenous immunoglobulin G + prednisone + platelet transfusion	Intracranial hemorrhage
		Romiplostim 8μg/kg to 10μg/kg	
2013	12	Intravenous immunoglobulin + prednisone + platelet transfusion	Intracranial hemorrhage

Five years after her initial intracranial hemorrhage, our patient had two more episodes of recurrent intracranial hemorrhage within the span of a year, at which time treatment with more aggressive cytotoxic chemotherapy was considered. A recent publication had reported the successful use of bortezomib in the treatment of a patient with refractory thrombotic thrombocytopenic purpura (TTP) in whom rituximab had been unsuccessful [2]. TTP has similar pathophysiology to ITP in that both disorders are antibody mediated. We therefore surmised that this agent may also have activity in refractory ITP, and treated our patient with bortezomib 1.3mg/m^2^ subcutaneously twice a week. Six weeks later, her platelet counts still ranged between 11,000 cells/mm^3^ and 20,000 cells/mm^3^.

**Figure 1:**
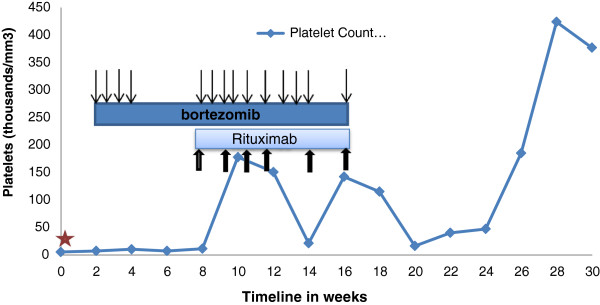
**Trend in platelet counts during treatment.** The y-axis depicts platelet counts in thousands/mm^3^ and the timeline is plotted on the x-axis. The star indicates the time of the intracranial bleed. The dark bar shows the duration of bortezomib treatment and arrows indicate timing of the injections; the lighter bar shows the duration of rituximab treatment with arrows indicating the timing of the treatments.

Our patient had received four out of 12 planned doses of bortezomib during this six weeks, primarily due to compliance issues. At this juncture, once-weekly intravenous rituximab (375mg/m^2^) was added to the regimen. Bortezomib was continued at the same dose twice weekly. Again, because of suboptimal compliance, she only received 10 out of 16 planned doses (Figure 1). There were no dose delays due to toxicity. This strategy was based on previously published data suggesting that these agents may be synergistic and that there may be a population of bortezomib-resistant B-cells that can be eliminated by the addition of rituximab [3,4]. Following this, our patient’s platelet count improved. Over the next three months, after some fluctuations, her platelet count normalized despite cessation of all therapy after six doses of rituximab. By this time, she had received a total of 14 subcutaneous injections of bortezomib (Figure 1). She did develop grade 1 neuropathy during the course of that treatment but otherwise tolerated it well. Her platelet count remained well above 100,000 cells/mm^3^ with the exception of three counts, one of which was below 40,000 cells/mm^3^, for 14 weeks of follow-up after the cessation of all therapy (Figure 1).

## Conclusion

To the best of our knowledge, this is the first report of the successful use of a combination of bortezomib and rituximab in a patient with highly refractory ITP. Our experience appears to be in line with the observations of Shortt *et al*. in the setting of another antibody-mediated disease, refractory TTP [2]. We believe our report strengthens the rationale for further study of this regimen in difficult cases of antibody-mediated autoimmune disorders such as TTP and ITP. It would also be an easier, less toxic alternative to conventional therapies in this setting, particularly with the anticipated availability in the near future of oral proteasome inhibitors that may have a lower risk of neuropathy as compared to bortezomib.

## Consent

Written informed consent was obtained from the patient for publication of this case report and any accompanying images. A copy of the written consent is available for review by the Editor-in-Chief of this journal.

## Abbreviations

ITP: Immune thrombocytopenia; TTP: Thrombotic thrombocytopenic purpura.

## Competing interests

The authors declare they have no competing interests.

## Authors’ contributions

All authors analyzed and interpreted the patient data and contributed in writing the manuscript. All authors read and approved the final manuscript.
